# Appearance quality, nutritional value, and aroma components of wild diguo (*Ficus tikoua* Bur.) fruit collected from southwest China

**DOI:** 10.1002/fsn3.4106

**Published:** 2024-03-19

**Authors:** Yang Li, Xu Yan, Juncheng Hu, Zizhou Wu, Zhouhe Du, Honglin Wang, Yanchun Zuo

**Affiliations:** ^1^ School of Urban‐Rural Planning and Construction Mianyang Teachers' College Mianyang China; ^2^ Institute of Special Economic Animals and Plants Sichuan Academy of Agricultural Sciences Nanchong China; ^3^ Forage Crops Germplasm Innovation and Production Management Key Laboratory of Nanchong City Nanchong China

**Keywords:** amino acid, aroma compound, crude protein, *Ficus tikoua*, geographical origin, sugar

## Abstract

Diguo (*Ficus tikoua* Bur.), an ancient wild fruit, is widely spread in southwest China. However, there is little information on the phenotypic traits, quality characteristics, and aroma compounds available to diguo fruit. The present study is an investigation into the effects of geographical origin on the phenotypic traits and quality characteristics of wild diguo fruit collected from southwest China. The volatile compounds in the mixed fruit samples were also investigated using gas chromatography–mass spectrometry. Our results indicated that significant variation existed among the sampling materials in all the phenotypic parameters. Fruit fresh weight ranged between 2.06 and 4.59 g. Moreover, significant variation existed among the selected materials in all macronutrients (dry matter, total soluble solids, crude protein, crude fat, and ash) and some nutritional parameters (glutamate, arginine, total soluble solids, maltose, and mannose, etc.). Regardless of their geographical origin, diguo fruit is relatively low in fat and fructose and high in fiber and glutamate. A total of 95 volatile constituents were identified in the frozen diguo fruit. In conclusion, diguo fruit with rich nutritional attributes has a promising future for commercial‐scale production. The variability of the observed morphological and nutritional features of diguo fruit provides important characteristics for improving the breeding of diguo as a modern fruit crop.

## Graphical abstract

Diguo fruits with rich nutritional attributes have a promising future for commercial‐scale production. The variability of the observed morphological and nutritional features of diguo fruits provides important characteristics for improving the breeding of diguo as a modern fruit crop.

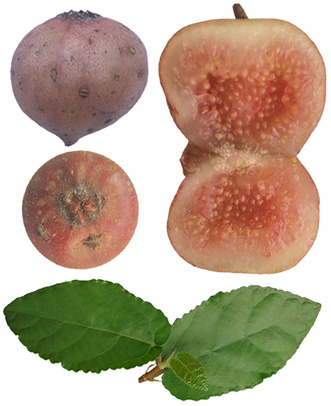

## 1 INTRODUCTION

*Ficus tikoua* Bureau, commonly known as “diguo” in China, is a creeping perennial woody vine of the *Ficus* genus in the family Moraceae. According to the *Flora of China*, this plant is native to southern China, India, Vietnam, and Laos. It can be found growing on roadsides, riversides, sandy hillsides, wastelands, rock crevices, and in open woodlands. Diguo is a versatile plant (Chen et al., 2023). As a traditional Chinese medicine, the entire plant has been used to treat various ailments, including cough, sore throat, chronic bronchitis, dyspepsia, dysentery, diarrhea, jaundice, edema, mastadenitis, rheumatism, impetigo, etc. (Bervinova et al., 2022; Yao et al., 2022). It is also cultivated for ornamental, groundcover, and bio‐ecological restoration purposes due to its adaptability, strong asexual reproductive capacity, and potential ability to absorb heavy metals (Chen et al., 2023).

Moreover, it is used as an edible plant that bears globular edible figs when ripe. In rural areas of southwestern China, people have fond memories of the diguo flavor. Due to its rich aroma and sweet taste, diguo fruit brings joy to people, making it particularly popular among young children and adolescents in the countryside. The wild diguo fruit, which is usually eaten fresh, is sold at local markets and on e‐commerce platforms. The price is reported to be up to 60 ~ 100 RMB (Renminbi) per kilogram (Cui et al., 2020; Wang et al., 2020). In 2021, we inquired about the price of diguo fruit on Taobao.com. The unit price (RMB/kg) of this fruit ranges from 213.80 to 398.08, with an average of 330. This implies that it is a fruit of significant economic value.

Previous studies have also shown that wild diguo fruit is rich in minerals. Compared to fig fruit, diguo fruit contains higher levels of K (4.33 vs. 2.12 mg/kg), Mg (452.4 vs. 170.0 mg/kg), Fe (38.5 vs. 1.0 mg/kg), and Ca (726.3 vs. 670.0 mg/kg) (Shi et al., 2012). Diguo fruit is low‐fat and low‐sodium (10.1 mg/kg DM) (Shi et al., 2012). Naturally, the consumption and processing methods of the diguo fruit can refer to figs. Diguo fruit can be eaten fresh or dried. Like figs (Barolo et al., 2014), they can also be processed into juices, paste, pulp, concentrate, nuggets, powder, or diced forms. Through processing, the economic value of the fruit can be further enhanced.

Although existing data suggests that diguo contains rich nutrients and active ingredients. However, the description of its fruit weight and size is limited and incomplete (Gong et al., 2022; Wang et al., 2021). Especially the differences in fruit from different regions are still unknown. Previous studies have primarily focused on the types and content of bioactive ingredients, with relatively little attention paid to the main nutritional components. Additionally, diguo fruit is found to be rich in dietary fiber and lysine, which cannot be synthesized in human and animal bodies. Therefore, diguo has the potential to be developed into a health food. Studies have shown that the diguo has higher levels of protein, carbohydrates, vitamin B2, vitamin C, vitamin E, magnesium, zinc, and other mineral elements, as well as the essential amino acids methionine and valine, compared to apples (Shi et al., 2012). The fruit has a higher protein content compared to figs, and its dietary fiber, carbohydrate, and carotene contents are also higher than those of apples, cherries, citrus, and other common fruits (Shi et al., 2012).

Considering the Data S1 on the potential nutraceutical properties of diguo, it is of interest to characterize the wild diguo that grows in southwest China. Accordingly, the objectives of this study were to determine the appearance quality, crude protein and amino acid contents, fiber, and carbohydrates contents of diguo from different geographic regions and to compare them with reported compositions for other reports.

## 2 MATERIALS AND METHODS

### 2.1 Plant materials

Wild fresh ripe figs of diguo were collected from different areas of Southwest China, namely, Neijiang, Dazhou, Nanchong, and Bazhong City in Sichuan Province, Liupanshui in Guizhou Province, and Liangping County in Chongqing City, from the middle of July to the middle of August 2021 (Table 1).

**TABLE 1:** Samples of wild diguo figs of different origins.

Access No.	Collecting time	Locality
LY01	Jul. 14, 2021	Dongxing District, Neijiang City, Sichuan Province
LY03	Jul. 14, 2021	Dachuan District, Dazhou City, Sichuan Province
LY06	Jul. 16, 2021	Peng'an County, Nanchong City, Sichuan Province
LY08	Jul. 28, 2021	Bazhou District, Bazhong City, Sichuan Province
LY10	Aug. 1, 2021	Liangping County, Chongqing
LY11	Aug. 2, 2021	Liuzhi Special Zone, Liupanshui City, Guizhou Province
LY14	Aug. 13, 2021	Gaoping District, Nanchong City, Sichuan Province

### 2.2 Appearance quality testing

The fruits were washed to remove the soil and dried with absorbent paper. Twenty intact fruits were randomly selected from each region, and the fruit width (diameter, mm) and length (mm) were measured using a Vernier caliper (Shanghai Menet MNT‐150) to calculate the fruit shape index by dividing the width by length. An electronic balance (Zhejiang Sunyu FA224) with 0.0001 g precision was used to measure the fresh weight of each individual fruit (unit mass). The moisture content (%FW) per fruit was then measured using an electric thermostatic blast dryer (Shanghai Xinmiao DHG‐9053A) at 65°C. Total soluble solids (%) was determined using a hand‐held refractometer (HB‐113ATC, Yuanhengtong) according to the standard NY/T 2637–2014.

### 2.3 Quality assessment

Three biological replicates, each comprised of a pool of fifty fruits, were sampled for each treatment. Samples for quality assessment were dried at 65°C to a constant weight. The dried fruits were crushed into a fine powder by mortar and pestle, enclosed in self‐sealing polythene bags, and stored at −20°C. Fiber (i.e., neutral detergent fiber) and crude fat were measured using the Ankom filter bag technique (Ankom Technology Corp). The determination of ash content was expressed as the percentage of residue remaining after dry oxidation in a Muffle furnace at 550°C for 6 h. The Kjeldahl method was applied to measure the total nitrogen (*N*) content (crude protein = 6.25 × total *N*) (Hanon K9860). According to the standard GB 5009.124–2016, the amino acid (without tryptophan and cystine) content was determined by an A300 automatic amino acid analyzer (membraPure). The mixed standard solution of amino acids (100 nmol/L) was analytically pure and purchased from Sinopharm Chemical Reagent Co. Ltd. These nutritional parameters were expressed on a dry‐weight basis.

A Waters Alliance 2695 HPLC was used for sugar determination. Weigh approximately 0.5 g of the dry sample (with an accuracy of 0.0001 g). Place it into a 100 mL triangular flask with a stopper. Add 50 mL of extraction solution (0.02 mol/L sodium hydroxide solution) accurately. Shake the flask at a speed of 180 r/min for 40 min. Filter the mixture using qualitative filter paper. Take 2 mL of the filtrate and filter it again using a 0.45 μm aqueous phase filter membrane. Finally, analyze the filtered solution. All sugar standards (e.g., glucose, sucrose, and maltose) were purchased from Sigma. The analysis column is Shodex's Asahipak NH2P‐50 4E normal phase chromatography column (with a column length of 150 mm, an inner diameter of 4.6 mm, and a fixed phase particle size of 5 μm). The mobile phase consists of water and acetonitrile, with a flow rate of 0.8 mL/min and an injection volume of 10 μL. The gradient elution is performed at a column temperature of 35°C. The detector is an evaporative light scattering detector, and the detection conditions are as follows: nitrogen as carrier gas, 500 gain, cooling as spray mode, drift tube temperature of 50°C, and gas pressure of 40 psi. The test duration is 30 min.

### 2.4 Aroma compound analysis

An Agilent 7890A GC coupled with a 5975C MSD was used for the determination of aroma compounds. The 1 g mixed frozen sample from seven different geographical sources was added to a 22 mL headspace bottle and heated at 60°C for 30 min. After solid‐phase microextraction, it was analyzed at 280°C for 5 min. Chromatographic conditions: the capillary column used was DB‐5MS (30 m × 0.25 mm × 0.25 μm) (J & W Scientific). The mobile phase employed was high‐purity helium (99.999%), with a flow rate of 1 mL/min. In non‐shunt mode, the inlet temperature is 250°C. The heating procedure for the column temperature box is as follows: maintain at 60°C for 0 min, then increase to 180°C at a rate of 5°C per minute, maintain for 1 min, and then rise to 240°C at a rate of 20°C per minute and maintain for 2 min. Quantitative analysis involves calculating the percentage of the peak area of the identified component to the area of the internal standard component, multiplied by the mass of the internal standard. This calculation provides a quantitative result, and the formula is as follows:

$$C_{i}=\frac{A_{i}}{A_{1}}\times m$$

where Ci – the content of an identified ingredient, μg; *A*_*i*_ – the peak area of an identified component; *A*_*1*_ is the internal standard peak area; *m* – internal standard quality.

### 2.5 Statistical analyses

Statistical analyses and graphical presentations were performed using R (R Core Team, 2023). The numbers of samples (*n*) used in the experiments are given in the table or figure. The significance of differences in multiple comparisons was determined using the Tukey test. The significance level was set at *p* < .05. Quantitative data were expressed as mean values with or without the respective standard deviation (mean ± SD).

## 3 RESULTS

### 3.1 Phenotypic traits

**FIGURE 1:**
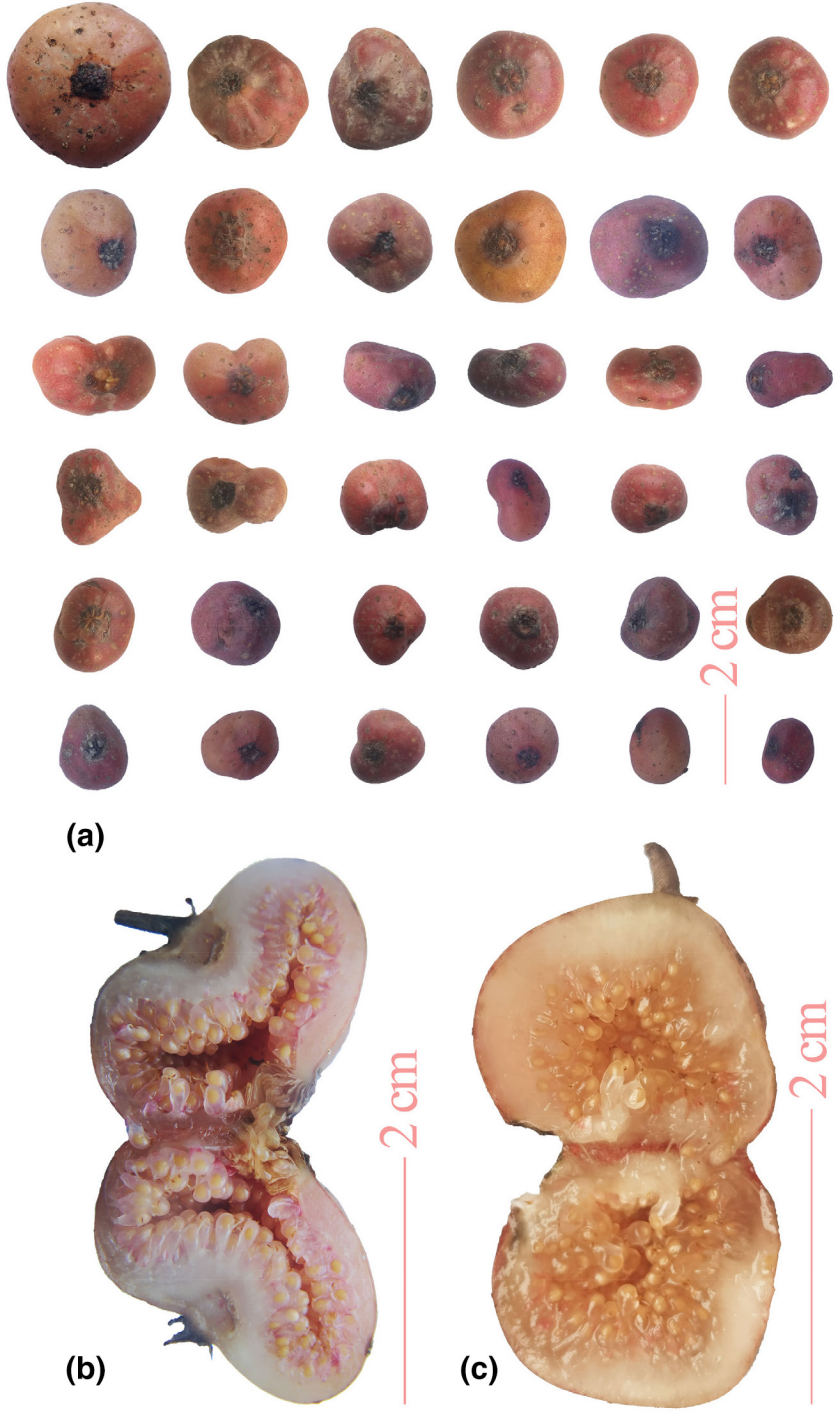
Phenotype of wild diguo fruit collected from southwest China. (a): Morphological diversity. (b): The fruit collected from Liuzhi Special Zone, Liupanshui City, Guizhou Province. (c): The fruit collected from Gaoping District, Nanchong City, Sichuan Province.

The color of mature diguo fruit ranges from light red to dark red (Figure 1a). Most diguo fruits have a spherical torus shape, with small holes located in the middle of the fruit. Studies have also shown that the color of ripe diguo ranges from light red to dark red. The shape of the diguo is usually a regular sphere with a small hole located in the middle. At the same time, irregular shapes such as heart‐shaped, gourd‐shaped, and kidney‐shaped were also observed (Figure 1a). The holes were not located in the geometric center of the diguo, indicating that the shape of the diguo may be easily influenced by its environment. These results indicate that the presence of hard objects, such as rocks and gravel, in the soil can contribute to the formation of fruit deformities. In addition, this study also revealed that there were two types of diguo pulp. The diguo fruit from Liupanshui in Guizhou showed no significant juice seepage and had larger seeds (Figure 1b), while diguo fruit from other geographical origins exhibited oozing flesh and smaller seeds (Figure 1c).

The fresh fruit weight of the diguo is shown in Table 2. The fruit fresh weight of diguo varied across seven different geographical sources, ranging from 1.1878 g to 7.6055 g. The average weight was 3.1002 g. Among these sources, LY11 had the highest fresh weight, with an average of 4.5865 g, which was significantly greater than the weight of the other materials. The average fresh weight of LY14 was 2.6242 g, which was significantly lower than that of the other materials. The fruit fresh weight of LY10 was more uniform compared to other materials, with the smallest coefficient of variation (19.93%). On the other hand, LY14 had the largest range of fruit weights, with a coefficient of variation of 42.91%.

**TABLE 2:** Fruit fresh weight of wild diguo collected from southwest China.

Access No.	Sample size	Min (g)	Max (g)	Mean (g)	SD	CV (%)
LY01	20	2.0708	4.5859	3.4058	0.7104	20.86
LY03	20	1.8786	4.9973	2.8994	0.7791	26.87
LY06	20	1.7311	5.0014	3.0405	0.8264	27.18
LY08	20	1.1878	3.7716	2.0570	0.7111	34.57
LY10	20	2.0201	4.2203	3.0876	0.6153	19.93
LY11	20	2.3843	7.6055	4.5865	1.5522	33.84
LY14	20	1.6022	5.4755	2.6242	1.1260	42.91
**All**	**140**	**1.1878**	**7.6055**	**3.1002**	**1.1814**	**38.11**

*Note*: Bold values indicate descriptive statistics for all tested samples.

The fruit shape index is shown in Table 3. The shape index of diguo varied across seven different geographical sources, ranging from 1.1127 to 1.3140. The average shape index was 1.1968. Among these materials, LY13 had the highest fruit shape index, with an average of 1.1372, which was significantly greater than the shape index of the other materials. The average shape index of LY03 was 1.1127, which was lower than that of the other materials. The shape index of LY14 was more uniform compared to other materials, with the smallest coefficient of variation (8.62%). On the other hand, LY11 had the largest range of fruit weights, with a coefficient of variation of 17.28%.

**TABLE 3:** Fruit shape of wild diguo collected from southwest China.

Access No.	Sample size	Min	Max	Mean	SD	CV (%)
LY01	20	0.9979	1.7185	1.2610	0.1768	14.02
LY03	20	0.8458	1.5342	1.1127	0.1606	14.44
LY06	20	0.9748	1.3864	1.1633	0.1359	11.68
LY08	20	0.9618	1.4711	1.1372	0.1283	11.29
LY10	20	1.0704	1.5816	1.1984	0.1287	10.74
LY11	20	0.9901	1.7767	1.3140	0.2270	17.28
LY14	20	0.9901	1.3747	1.1912	0.1027	8.62
**All**	**140**	**0.8458**	**1.7767**	**1.1968**	**0.1661**	**13.88**

*Note*: Bold values indicate descriptive statistics for all tested samples.

### 3.2 Quality characteristics

**FIGURE 2:**
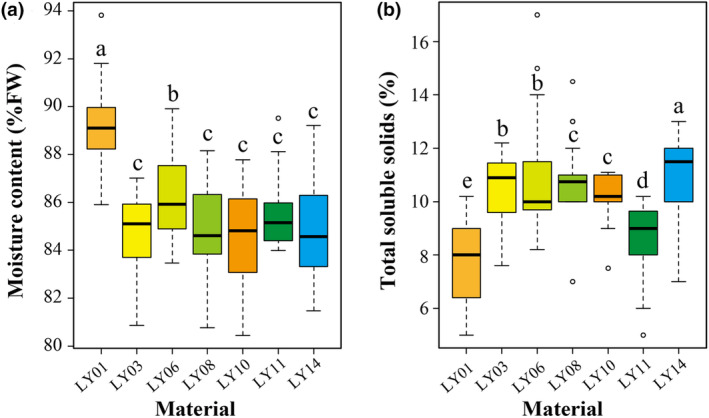
Moisture content (a) and total soluble solids (b) of wild diguo fruit collected from southwest China. Different letters indicate significant differences (*p* < .05) in each constituent (sic *passim*).

**FIGURE 3:**
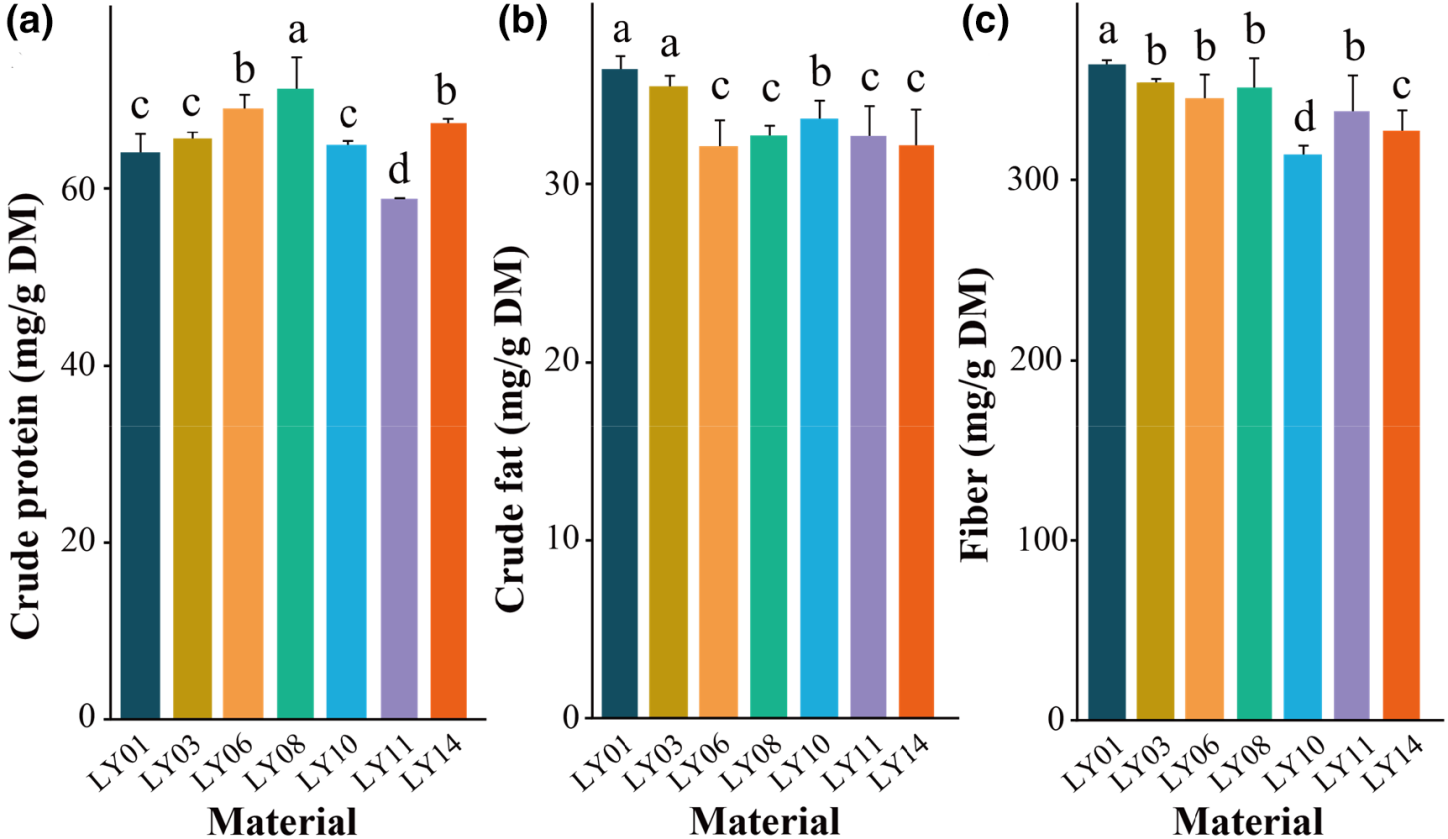
Macronutrients in wild diguo fruit collected from southwest China.

Among all the fruit surveyed, the water content varied from 80.45% to 93.82% FW, with an average value of 85.68% FW (Table S1). The range of total soluble solids is 5.0% ~ 17.0%, with an average value of 9.99% (Table S2). The crude protein content varies from 58.69 to 74.87 mg/g DM, with an average of 65.89 mg/g DM (Table S3). The crude fat content ranges from 28.90 to 37.20 mg/g DM, with an average of 33.62 mg/g DM (Table S4). The range of fiber content is 308.0 ~ 367.7 mg/g DM, with an average value of 338.6 mg/g DM (Table S5). The range of crude ash content is 62.5 ~ 106.6 mg/g DM, with an average value of 77.2 mg/g DM (Table S6). The comparison of the main nutritional components of fruit from different geographical sources (Figures 2 and 3 and Tables S1–S6) shows that LY01 has a higher moisture content and crude fat content and lower total soluble solids compared to other materials. Additionally, LY08 has the highest protein content (71.33 mg/g DM). LY011 exhibits the lowest protein content (58.83 mg/g DM), as well as lower total soluble solids and crude fat content.

**FIGURE 4:**
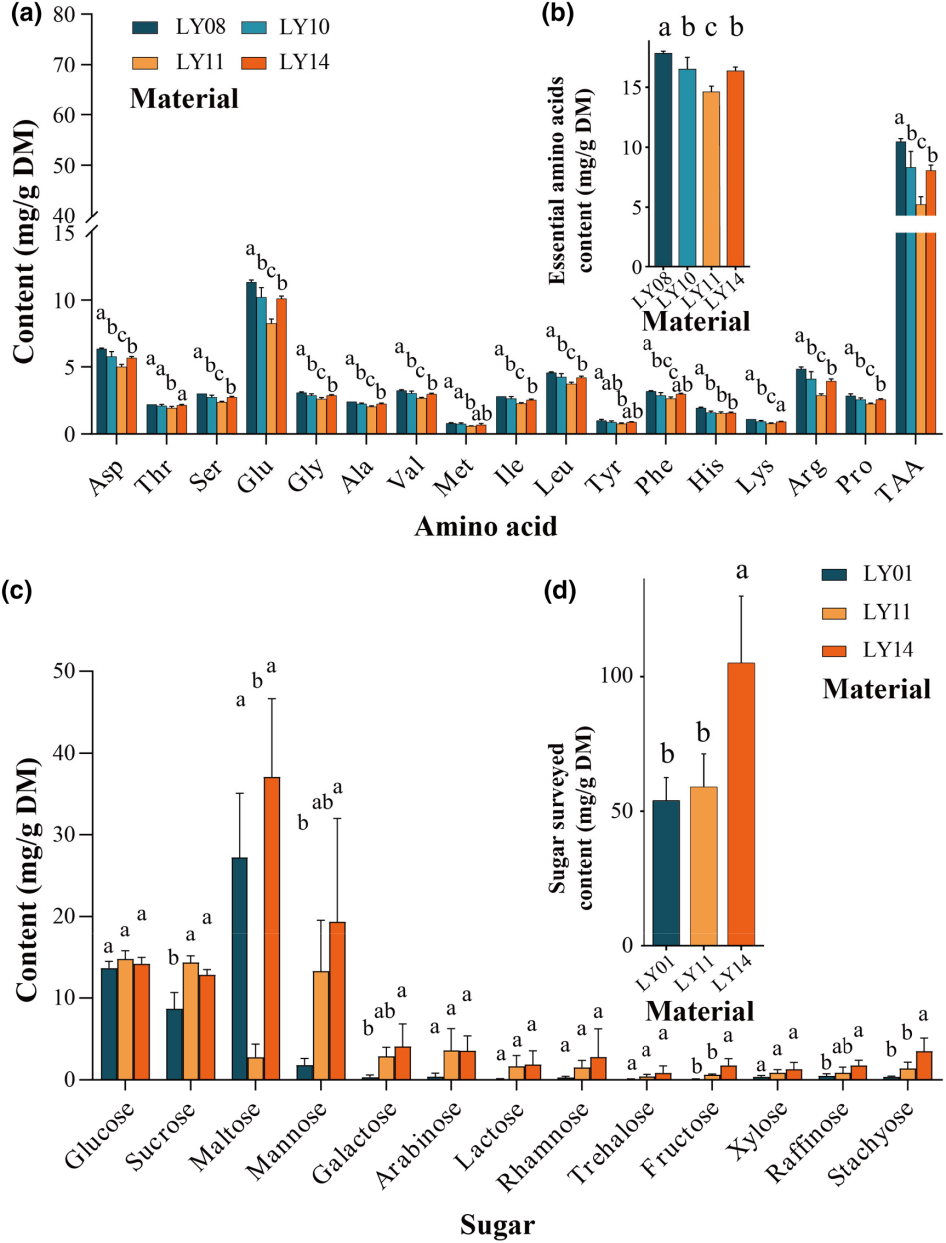
Amino acid (a) and sugar (b) composition of wild diguo fruit collected from southwest China.

Furthermore, we analyzed the amino acid composition of four materials that exhibited significant differences in crude protein content, namely LY08, LY10, LY11, and LY14 (Figure 4a and Table S8). Amino acid analysis indicates that the total amino acid content increases with an increase in crude protein content. The proportion of total amino acid content to crude protein content varies from 71.78% to 76.67%, with an average of 74.34%. The range of essential amino acid content is 14.61–17.87 mg/g DM, with an average of 16.36 mg/g DM. LY08 contains the highest content of essential amino acids, followed by LY10 and LY14, while LY11 has the lowest essential amino acid content (Figure 4b). The proportion of essential amino acids to total amino acids varies from 32.69% to 34.60%, with an average of 33.54%. Among the essential amino acids, leucine has the highest content (3.73–4.57 mg/g DM), followed by valine, phenylalanine, isoleucine, threonine, lysine, and methionine. This trend was consistent across all materials surveyed (Table S7). The range of nonessential amino acid content is 27.62–36.80 mg/g DM, with an average of 32.50 mg/g DM. Among nonessential amino acids, glutamic acid content is the highest (8.27–11.33 mg/g DM), followed by aspartic acid, arginine, glycine, serine, proline, alanine, histidine, and tyrosine. This trend was consistent across all the materials surveyed (Table S7).

The sugar content of LY01, LY11, and LY14 was analyzed. The sugar content of LY14 (104.94 mg/g DM) was significantly higher than that of LY01 (53.85 mg/g DM) and LY11 (59.05 mg/g DM) (Figure 4b). There were no significant differences in the levels of glucose, arabinose, lactose, rhamnose, trehalose, and xylose among the materials surveyed. However, other sugars exhibited significant variations among the materials surveyed (Figure 4c and Table S8). Overall, glucose, maltose, sucrose, and mannose are the most prevalent sugars in the diguo fruit, constituting 76.49% to 95.38% of the total sugar content. However, maltose and mannose show variations in content between materials. The maltose content in LY11 (2.78 mg/g DM) was significantly lower than that in LY01 and LY14 (27.22 and 37.11 mg/g DM). The mannose content of LY01 (1.81 mg/g DM) is significantly lower than that of LY14 (19.35 mg/g DM). Diguo fruit has a lower fructose content (0.10–1.77 mg/g DM), and the fructose content of LY14 is significantly higher than that of the other two materials.

### 3.3 Aroma compounds

Through sensory evaluation, it was found that LY11 has a lighter aroma, while the fruit aroma of the other materials is more intense. A total of 95 volatile substances were detected in the frozen diguo from a mixed sample of seven materials from southwest China. The volatile substances in diguo are primarily composed of seven types of compounds, including alcohols, esters, aldehydes, ketones, phenols, acids, and other compounds (Table S9). Esters are one of the main components that form the aroma of diguo fruit. These volatile substances and their interactions contribute to the distinctive aroma of diguo. Some aroma compounds that have been used in the food industry include 2‐undecanone, ethyl hexanoate, ethyl vanillin, longifolene, phenylethyl alcohol, lilial, etc. Among these aroma substances, the 10 components with the highest relative content are as follows: sebacic acid, 2‐ethylhexyl octyl ester; benzene, 1,1′‐[1,2‐ethanediylbis(oxymethylene)]bis; butylphosphonic acid, diphenyl ester; beta‐neoclovene; cyclo‐(l‐leucyl‐l‐phenylalanyl); trans‐13‐docosenamide; ethyl vanillin; acetic acid, 2‐phenylethyl ester; dibutyl phthalate; and phenoxyethanol, TMS derivative.

## 4 DISCUSSION

### 4.1 Genetic and cultivation manipulation for improving diguo fruit size and quality

Fruit weight is an important parameter both for scientific understanding and for commercial consumption. The fruit fresh weight of wild figs ranges from 0.56 to 28.69 g in Iran, with an average of 8.33 g (Mirheidari et al., 2020). In a recent study, the evaluation of the fruit traits of 49 wild figs showed that their fruit length ranged from 12.65 to 22.60 mm, fruit width ranged from 10.67 to 24.18 mm, and fruit fresh weight ranged from 2.52 to 6.13 g (Khadivi & Mirheidari, 2022). The wild diguo fruit collected from rocky desertification areas in Guizhou has a fruit diameter of 1.79 cm and a fruit fresh weight of 2.76 g (Wang et al., 2021). Here, the fresh weight of the fruit ranged from 2.05 to 4.58 g (Table 2), slightly lower than the fresh weight of wild fig fruit (Khadivi & Mirheidari, 2022). In the studies of fig cultivars, the fruit fresh weights ranged from 24.5 to 49.0 g (Abdelsalam et al., 2019), 29.97 to 59.88 g (Mahmoudi et al., 2018), and 34.54 to 96.45 g (Gaaliche et al., 2012). The changes in fruit size of wild and cultivated fig cultivars (a relative of diguo) mentioned above have given us confidence in enhancing the fruit size of diguo through breeding and cultivation techniques.

Although the diguo has shown potential for ecological restoration, garden cover, medicine, and food, there is currently no breeding work targeting a specific application of the diguo (Chen et al., 2023). As wild resources can be exploited, it is crucial to identify and select outstanding cultivars from various wild materials. Fortunately, chloroplast gene, nuclear gene (microsatellites) analyses, and AMOVA (analysis of molecular variance) indicate that the diguo populations are highly differentiated (Chen et al., 2011; Fu, 2018). This provides a germplasm basis for breeding different uses of diguo. For example, this study also found differences in sugar metabolism in diguo fruit. The mannose content of LY01 is lower (1.81 vs. 13.27–19.35 mg/g); the maltose content of LY11 is lower (2.78 vs. 27.22–37.11 mg/g). Like figs, the female plant of Diguo is the only producer of edible fruit. The breeding system of diguo is a complex mutualistic symbiosis between the plant and its pollinator, the wasp Ceratosolen (Yuan et al., 2021). The breeding methods of figs can be used as a reference in the breeding of diguo fruit. Further breeding with these resources may provide new cultivars with higher quality and diversity. To reach its full potential, more studies are needed.

Meanwhile, by drawing inspiration from crop breeding strategies, it is possible to rapidly combine multiple desirable genes using techniques such as hybridization, chromosome doubling, genomics, gene functional trait analysis, molecular marker‐assisted breeding, and gene editing. This allows for the rapid development of new fruit cultivars. Diguo is a prostrate shrub with figs partially buried in the soil (Chen et al., 2011), which makes fruit harvesting difficult. Moreover, hard objects in the soil can easily cause deformities in fruit. Off‐ground cultivation or grafting on closely related species, such as figs, to facilitate pollination and fruit ripening in the air may emerge as an alternative method to enhance the size and quality of diguo fruit.

### 4.2 Nutritional properties of diguo fruit

The total amino acid content (without tryptophane and cysteine) in the diguo fruit of Qinglong County, Guizhou Province was 89.99 mg/g DM, and the three amino acids with the highest amino acid content were valine (21.30 mg/g DM), threonine (12.00 mg/g DM), and glutamate (5.71 mg/g DM) (Shi et al., 2012). The ripe diguo fruit collected from Beichuan County, Sichuan Province, China has a relatively high crude protein content (94.1 mg/g DM) and total amino acid content (92.8 mg/g DM, without tryptophane), and the four prevalent amino acids were aspartic acid (14.8 mg/g DM), glutamate (14.0 mg/g DM), leucine (6.9 mg/g DM), and arginine (6.2 mg/g DM) (Gong et al., 2022). The trend of changes in amino acid content of the four materials in this study (Figure 4a) is similar to the results of (Gong et al., 2022), with glutamate content being the highest (8.27–11.33 mg/g DM), followed by aspartic acid (5.00–6.37 mg/g DM), leucine (3.73–4.57 mg/g DM), and arginine (2.87–4.83 mg/g DM). However, the total amino acid content (without tryptophane and cysteine) in this study varied from 42.20 to 54.70 mg/g, which was lower than the previous reports (89.99–90.7 mg/g DM) (Gong et al., 2022; Shi et al., 2012). The content of essential amino acids (14.61–17.87 mg/g DM) is also lower than previous reports (30.6–57.634 mg/g DM) (Table S7). Glutamate and aspartic acid have a delicious and delicate taste, which may partially explain the delicious taste of diguo fruit. At the same time, the presence of aroma components makes the flavor of the fruit pleasant.

When the fruit is ripe, the contents of glucose, fructose, and sucrose are 2.93, 3.08, and 16.16 mg/g, respectively, and the sucrose content is significantly higher than that of glucose and fructose at all stages of fruit development (Wang et al., 2021). A report showed that the sucrose content (16.5057–25.5161 mg/g) of three fresh fruit from Guiyang, Qinglong, and Majiang counties was significantly higher than that of fructose (2.3942–3.9466 mg/g) and glucose (1.5455–3.2863 mg/g) and the content varied in different regions (Yang et al., 2016). The determination of sugars in this study showed that the glucose (13.64–14.77 mg/g DM) and sucrose content (8.69–14.34 mg/g DM) values of diguo fruit from three regions were similar, but both were significantly higher than the fructose content (0.10–1.77 mg/g DM). Excessive consumption of fructose has been linked to visceral fat accumulation, hyperlipidemia, insulin resistance, hypertension, and hyperuricemia in humans (Shi et al., 2021). In a survey, the fructose content of 32 types of fruits surveyed varied from 23.08 to 391.33 mg/g DM, with an average of 162.98 mg/g DM (Jovanovic‐Malinovska et al., 2014) (Table S10). In summary, duguo fruit is relatively low in fructose, and it may be consumed by patients with fructose intolerance. Fructose can promote an increase in uric acid levels. Diguo may also be used as a healthy fruit for patients with high uric acid levels. Although diguo fruit contains sucrose (glucose:fructose = 1:1), a recent report has shown that the uric acid‐raising effect of sucrose is weaker than that of fructose (Kawakami et al., 2023). More research is needed to determine whether the diguo fruit can be safely consumed by patients with high uric acid levels and fructose intolerance.

Based on the results of this study and previous studies, it is indicated that the diguo fruit is a promising source of functional food. For instance, the diguo fruit also has a high polyphenol content (8.6 mg/g DM), which could be an excellent source of antioxidants (Gong et al., 2022). The pomological characteristics and nutritional value of diguo fruit may be influenced by various physiological and environmental factors, including soil chemistry, altitude, slope, vegetation, age, and climatic conditions. In addition to the environmental factor, the genetic diversity resulting from the geographical isolation of the diguo may play a significant role in the variations in the nutritional composition of the fruit. For example, high genetic differentiation was observed between two populations that were only 31 km apart (Chen et al., 2011). In China, diguo is grown all over the Southwest region. Further studies with fruit from plants collected from different localities but planted in the same orchard would provide more precise information on pomological characteristics and nutritional value.

### 4.3 roma of diguo fruit

The aroma of the diguo fruit is the result of a subtle mixture of volatile compounds, and it is a crucial factor for consumer acceptance. When the diguo fruit ripens, its delightful aroma can be smelled from several meters away. These flavors may help attract animals to feed, thereby aiding in the dispersal of the seeds. When gathering fruits in the wild, it is common to find ripe fruits that have been partially eaten. The aroma components are influenced by multiple factors, including genotype, maturity period, storage conditions, and detection conditions. This study only conducted a preliminary investigation of the aroma components of frozen diguo fruit. The frozen diguo fruit still has a fragrance, but the aroma weakens after refrigeration. It is necessary to further analyze which substances are absent and causing the flavor to diminish. According to sensory evaluation, the richness of the fruit's aroma varies among different materials, suggesting that it is possible to select fruit with different types of aromas.

The volatile compounds in diguo fruit are relatively abundant. In bananas, 62 and 59 volatile components were detected in Fenjiao (*Musa* ABB Pisang Awak) and Brazilian fruits (*Musa* spp. AAA group), respectively (Zhu et al., 2018). The number of aroma compounds in ‘Shine Muscat’ grapes varies from 23 to 36 during different harvesting periods (Wei et al., 2022). A total of 38 volatile aroma components were detected in fresh *Prunus salicina* L. cv ‘Friar’ (Wang et al., 2018). We identified 95 types of volatile compounds in diguo fruits, and fresh diguo fruit may contain a greater quantity and concentration of aroma compounds. We speculate that the diguo fruit may be a potential candidate for the development of food essences.

## 5 CONCLUSION

It is seen that *Ficus tikoua* is a valuable edible fruit plant due to its nutraceutical features, which include rich protein and fiber, a pleasant taste, and aroma. Wild diguo fruit is rich in amino acids and sugars, predominantly glutamate, leucine, arginine, glucose, and sucrose. Moreover, it is relatively low in fat and fructose. Based on these phytochemical attributes, we recommend diguo fruit as a promising fruit crop of nutritional and economic importance. Some nutrient compositions of diguo fruit were significantly different in various regions, indicating that the fruit quality is influenced by the specific production location or genetic composition. Further research should, therefore, be conducted on diguo fruit and its breeding, and the nutraceutical results of diguo fruit should be disseminated to the public.

## AUTHOR CONTRIBUTIONS

**Yang Li:** Conceptualization (lead); project administration (lead); resources (lead); supervision (lead); writing – review and editing (lead). **Xu Yan:** Data curation (equal); methodology (equal); visualization (lead); writing – original draft (equal). **Juncheng Hu:** Data curation (lead); methodology (equal). **Zizhou Wu:** Data curation (equal); methodology (equal); validation (equal). **Zhouhe Du:** Data curation (equal). **Honglin Wang:** Data curation (equal); validation (equal). **Yanchun Zuo:** Data curation (supporting).

## CONFLICT OF INTEREST STATEMENT

The authors declare that there are no conflicts of interest.

## Supporting information


Data S1.

## Data Availability

The authors confirm that the data supporting the findings of this study are available within the article [and/or its supplementary materials].
