# Supplementary material for: Appearance quality, nutritional value, and aroma components of wild diguo (Ficus tikoua Bur.) fruit collected from southwest China
Source: Food Sci Nutr. 2024 Mar 19;12(6):4399–407. doi: 10.1002/fsn3.4106 (PMC11167135; doi:10.1002/fsn3.4106)
Supplement: Supplementary file 1 — Data S1. [file FSN3-12-4399-s001.docx]

**Supplementary Information for**

## Appearance quality, nutritional value, and aroma components of wild diguo (*Ficus tikoua* Bur.) fruit collected from southwest China

Yang Li^1*^ | Xu Yan^2,3^ | Jun-cheng Hu^1^ | Zi-zhou Wu^2^ | Zhou-he DU^2^ | Hong-lin Wang^2^ | Yan-chun Zuo^2^

^1^School of Urban-Rural Planning and Construction, Mianyang Teachers’ College, Mianyang, China

^2^Institute of Special Economic Animals and Plants, Sichuan Academy of Agricultural Sciences, Nanchong, China

^3^Forage Crops Germplasm Innovation and Production Management Key Laboratory of Nanchong City, Nanchong, China

^*^Correspondence: Yang Li, School of Urban-Rural Planning and Construction, Mianyang Teachers’ College, Mianyang, China.

E-mail: [yang_li@mtc.edu.cn](mailto:yang_li@mtc.edu.cn)

This file includes:

TABLES S1 to S10

FIGURES S1 to S2

SI REFERENCES

TABLE S1 Moisture content of wild diguo fruit collected from southwest China.

| **Access No.** | **Sample size** | **Min*** | **Max*** | **Mean*+** | **SD** | **CV (%)** |
| --- | --- | --- | --- | --- | --- | --- |
| LY01 | 20 | 85.91 | 93.82 | 89.24a | 1.7074 | 1.92 |
| LY03 | 20 | 80.86 | 87.02 | 84.78c | 1.5455 | 1.83 |
| LY06 | 20 | 83.46 | 89.90 | 86.20b | 1.7206 | 2.00 |
| LY08 | 20 | 80.77 | 88.16 | 84.79c | 1.9904 | 2.35 |
| LY10 | 20 | 80.45 | 87.78 | 84.41c | 2.0264 | 2.40 |
| LY11 | 20 | 83.98 | 89.51 | 85.57c | 1.4733 | 1.72 |
| LY14 | 20 | 81.47 | 89.21 | 84.77c | 2.0311 | 2.39 |
| **All** | **140** | **80.45** | **93.82** | **85.68** | **2.3529** | **2.75** |

*: (%FW).

**+**: Different letters indicate significant differences (*P*<0.05) in column (*sic passim*).

TABLE S2 Total soluble solids of wild diguo fruit collected from southwest China.

| **Access No.** | **Sample size** | **Min (%)** | **Max (%)** | **Mean (%)** | **SD** | **CV (%)** |
| --- | --- | --- | --- | --- | --- | --- |
| LY01 | 20 | 5.0 | 10.2 | 7.73e | 1.4743 | 19.07 |
| LY03 | 20 | 7.6 | 12.2 | 10.50b | 1.2390 | 11.80 |
| LY06 | 20 | 8.2 | 17.0 | 10.90b | 2.2458 | 20.60 |
| LY08 | 20 | 7.0 | 14.5 | 10.90c | 1.5140 | 13.89 |
| LY10 | 20 | 7.5 | 11.1 | 10.20c | 0.8756 | 8.58 |
| LY11 | 20 | 5.0 | 10.2 | 8.60d | 1.4200 | 16.51 |
| LY14 | 20 | 7.0 | 13.0 | 11.10a | 1.5549 | 14.01 |
| **All** | **140** | **5.0** | **17.0** | **9.99** | **1.9226** | **19.25** |

TABLE S3 Crude protein content of wild diguo fruit collected from southwest China.

| **Access No.** | **Sample size** | **Min*** | **Max*** | **Mean*** | **SD** | **CV (%)** |
| --- | --- | --- | --- | --- | --- | --- |
| LY01 | 3 | 60.62 | 66.15 | 64.05c | 2.9988 | 4.68 |
| LY03 | 3 | 64.89 | 66.36 | 65.57c | 0.7410 | 1.13 |
| LY06 | 3 | 66.96 | 70.64 | 69.08b | 1.9051 | 2.76 |
| LY08 | 3 | 69.02 | 74.87 | 71.33a | 3.1126 | 4.36 |
| LY10 | 3 | 64.07 | 65.35 | 64.91c | 0.7257 | 1.12 |
| LY11 | 3 | 58.69 | 58.90 | 58.83d | 0.1184 | 0.20 |
| LY14 | 3 | 66.94 | 67.94 | 67.44b | 0.5026 | 0.75 |
| **All** | **21** | **58.69** | **74.87** | **65.89** | **4.1032** | **6.23** |

*: (mg/g DM).

TABLE S4 Crude fat content of wild diguo fruit collected from southwest China.

| **Access No.** | **Sample size** | **Min*** | **Max*** | **Mean*** | **SD** | **CV (%)** |
| --- | --- | --- | --- | --- | --- | --- |
| LY01 | 3 | 36.00 | 37.20 | 36.47a | 0.6457 | 1.77 |
| LY03 | 3 | 35.00 | 36.10 | 35.51a | 0.5789 | 1.63 |
| LY06 | 3 | 30.30 | 33.60 | 32.11c | 1.6789 | 5.23 |
| LY08 | 3 | 31.70 | 33.30 | 32.71c | 0.8828 | 2.70 |
| LY10 | 3 | 33.20 | 34.70 | 33.70b | 0.8943 | 2.65 |
| LY11 | 3 | 31.70 | 34.40 | 32.68c | 1.4960 | 4.58 |
| LY14 | 3 | 28.90 | 34.20 | 32.16c | 2.8210 | 8.77 |
| **All** | **21** | **28.90** | **37.20** | **33.62** | **2.0521** | **6.10** |

*: (mg/g DM).

TABLE S5 Fiber content of wild diguo fruit collected from southwest China.

| **Access No.** | **Sample size** | **Min*** | **Max*** | **Mean*** | **SD** | **CV (%)** |
| --- | --- | --- | --- | --- | --- | --- |
| LY01 | 3 | 361.3 | 366.6 | 364.3a | 2.6958 | 0.74 |
| LY03 | 3 | 353.1 | 356.5 | 354.3b | 1.9132 | 0.54 |
| LY06 | 3 | 336.9 | 358.7 | 345.5b | 11.5743 | 3.35 |
| LY08 | 3 | 338.3 | 367.7 | 351.5b | 14.9388 | 4.25 |
| LY10 | 3 | 308.0 | 318.9 | 314.0d | 5.5264 | 1.76 |
| LY11 | 3 | 314.1 | 358.2 | 338.4b | 22.3682 | 6.61 |
| LY14 | 3 | 310.7 | 338.9 | 327.6c | 14.8403 | 4.53 |
| **All** | **21** | **308.0** | **367.7** | **338.6** | **19.5134** | **5.76** |

*: (mg/g DM).

TABLE S6 Ash content of wild diguo fruit collected from southwest China.

| **Access No.** | **Sample size** | **Min*** | **Max*** | **Mean*** | **SD** | **CV (%)** |
| --- | --- | --- | --- | --- | --- | --- |
| LY01 | 3 | 83.0 | 100.4 | 88.9a | 9.9301 | 11.17 |
| LY03 | 3 | 62.5 | 70.8 | 65.9e | 4.3692 | 6.63 |
| LY06 | 3 | 71.0 | 85.7 | 79.9c | 0.7750 | 0.97 |
| LY08 | 3 | 68.2 | 77.7 | 73.9d | 5.0474 | 6.83 |
| LY10 | 3 | 66.2 | 75.1 | 71.1d | 4.5149 | 6.35 |
| LY11 | 3 | 69.0 | 85.1 | 77.8c | 8.1612 | 10.49 |
| LY14 | 3 | 69.6 | 106.6 | 83.3b | 20.2836 | 24.35 |
| **All** | **21** | **62.5** | **106.6** | **77.2** | **10.6341** | **13.77** |

*: (mg/g DM).

TABLE S7 Amino acid content of wild diguo fruit collected from southwest China.

| **Amino acid** | **Access No.** | | | |  | **Other reports** | |
| --- | --- | --- | --- | --- | --- | --- | --- |
|  | **LY08** | **LY10** | **LY11** | **LY14** |  | **BC** | **QL** |
| Essential amino acids (mg/g DM) | | | | | | | |
| Leu | 4.57a | 4.23b | 3.73c | 4.20b |  | 6.9 | 2.292 |
| Val | 3.23a | 3.03b | 2.67c | 2.97b |  | 5.1 | 21.297 |
| Phe | 3.17a | 2.87bc | 2.63c | 2.97ab |  | 4.8 | 7.562 |
| Ile | 2.80a | 2.63b | 2.27c | 2.53b |  | 4.0 | 4.818 |
| Thr | 2.20a | 2.10a | 1.93b | 2.13a |  | 3.5 | 11.995 |
| Lys | 1.10a | 0.93b | 0.79c | 0.92a |  | 5.1 | 4.116 |
| Met | 0.80a | 0.75a | 0.59b | 0.68ab |  | 1.2 | 5.554 |
| Trp | -- | -- | -- | -- |  | -- | 3.414 |
| **EAA** | **17.87a** | **16.54b** | **14.61c** | **16.40b** |  | **30.6** | **57.634** |
| Nonessential amino acids (mg/g DM) | | | | | | | |
| Glu | 11.33a | 10.20b | 8.27c | 10.10b |  | 14.0 | 5.705 |
| Asp | 6.37a | 5.80b | 5.00c | 5.70b |  | 14.8 | 4.835 |
| Arg | 4.83a | 4.10b | 2.87c | 3.93b |  | 6.2 | 0.552 |
| Gly | 3.07a | 2.87b | 2.60c | 2.87b |  | 4.4 | 4.032 |
| Ser | 3.00a | 2.73b | 2.37c | 2.73b |  | 5.0 | 1.807 |
| Pro | 2.83a | 2.57b | 2.23c | 2.57b |  | 4.7 | 5.621 |
| Ala | 2.40a | 2.23b | 2.03c | 2.23b |  | 4.9 | 4.701 |
| His | 1.93a | 1.60b | 1.53b | 1.57b |  | 3.5 | 0.318 |
| Tyr | 1.00a | 0.88ab | 0.77b | 0.89ab |  | 2.4 | 4.785 |
| Cys | -- | -- | -- | -- |  | 2.1 | 4.283 |
| **NEAA** | **36.80a** | **33.03b** | **27.62c** | **32.53b** |  | **59.9** | **32.356** |
| **TAA** | **54.67a** | **49.57b** | **42.23c** | **48.93b** |  | **90.5** | **89.990** |

**BC**: The wild fruit was collected from Beichuan County, Sichuan Province, China (Shi et al., 2012)

**QL**: The wild fruit was collected from Qinglong County, Guizhou Province, China (Gong et al., 2022)

**--**: Not Available

**EAA**: Essential amino acids (without tryptophan)

**NEAA**: Nonessential amino acids (without cystine)

**TAA**: Total amino acids (without tryptophan and cystine)

Different letters indicate significant differences (*P*<0.05) in each row (*sic passim*).

TABLE S8 Sugar content of wild diguo fruit collected from southwest China.

| **Sugar** | **Sample size** | **Access No. (mg/g DM)** | | |
| --- | --- | --- | --- | --- |
|  |  | **LY01** | **LY11** | **LY14** |
| Glucose | 3 | 13.64a | 14.77a | 14.15a |
| Sucrose | 3 | 8.69b | 14.34a | 12.82a |
| Maltose | 3 | 27.22a | 2.78b | 37.11a |
| Mannose | 3 | 1.81b | 13.27ab | 19.35a |
| Galactose | 3 | 0.32b | 2.89ab | 4.09a |
| Arabinose | 3 | 0.39a | 3.60a | 3.56a |
| Lactose | 3 | 0.09a | 1.66a | 1.87a |
| Rhamnose | 3 | 0.31a | 1.53a | 2.79a |
| Trehalose | 3 | 0.08a | 0.41a | 0.84a |
| Fructose | 3 | 0.10b | 0.63b | 1.77a |
| Xylose | 3 | 0.36a | 0.87a | 1.30a |
| Raffinose | 3 | 0.48b | 0.88ab | 1.77a |
| Stachyose | 3 | 0.36b | 1.41b | 3.52a |
| Sugar surveyed | 3 | 53.85b | 59.05b | 104.94a |

TABLE S9 Aromatic compounds in wild diguo fruit collected from southwest China.

| **Number** | **Compound** | **Content (μg/kg)** |
| --- | --- | --- |
| 1 | Sebacic acid, 2-ethylhexyl octyl ester | 464.0359 |
| 2 | Benzene, 1,1'-[1,2-ethanediylbis(oxymethylene)] bis | 110.9110 |
| 3 | Butylphosphonic acid, diphenyl ester | 110.1843 |
| 4 | beta-Neoclovene | 103.8481 |
| 5 | Cyclo-(l-leucyl-l-phenylalanyl) | 88.1696 |
| 6 | trans-13-Docosenamide | 81.2487 |
| 7 | Ethyl Vanillin | 61.0422 |
| 8 | Acetic acid, 2-phenylethyl ester | 55.0000 |
| 9 | Dibutyl phthalate | 45.8219 |
| 10 | phenoxyethanol, TMS derivative | 42.9399 |
| 11 | Androstane-3,17-diol, 17-methyl-, (3.alpha.,5.alpha.,17.beta.) | 41.2878 |
| 12 | Phthalic acid, 2-cyclohexylethyl isobutyl ester | 33.2333 |
| 13 | Benzenamine, N-[(4-chlorophenyl)methylene]-, N-oxide | 30.3126 |
| 14 | Cyclopentanecarboxylic acid, 2-methylpropyl ester | 29.0992 |
| 15 | Glutaric acid, hexyl 2-propylpentyl ester | 22.0794 |
| 16 | Succinic acid, 5-methoxy-3-methylphenyl pentyl ester | 19.2851 |
| 17 | Glutaric acid, cyclohexylmethyl 3-fluorophenyl ester | 13.1330 |
| 18 | 4-Ethylbenzoic acid, ethyl ester | 7.7093 |
| 19 | Cyclotetrasiloxane, octamethyl | 6.5551 |
| 20 | Propanoic acid, 2-methyl-, 1,2,3-propanetriyl ester | 6.0545 |
| 21 | 2-(Trifluoromethyl)benzyl alcohol, picolinyloxydimethylsilyl ether | 5.0619 |
| 22 | Cyclopentasiloxane, decamethyl | 4.0052 |
| 23 | Cyclopentanecarboxylic acid, nonyl ester | 3.8610 |
| 24 | Phenylethyl Alcohol | 3.8253 |
| 25 | 4-Ethylbenzoic acid, 4-methylpentyl ester | 3.8078 |
| 26 | m-Toluic acid, undecyl ester | 3.3612 |
| 27 | Succinic acid, ethyl 2-methylphenyl ester | 2.5021 |
| 28 | 2-Ethyl-1-hexanol | 2.4616 |
| 29 | Nerolidol | 2.3915 |
| 30 | 4,6-Bis(diethylamino)-1,3,5-triazine-2-carbonylhydrazide | 2.1418 |
| 31 | 7,7-Dibromobicyclo[2.2.1]heptane-1-carboxylic acid, methyl ester | 2.0710 |
| 32 | 1-(4-Hydroxy-3-methoxyphenyl)oct-4-en-3-one | 2.0288 |
| 33 | Propanoic acid, 2-methyl-, 3-hydroxy-2,2,4-trimethylpentyl ester | 1.7734 |
| 34 | Carbamic acid, N-phenyl-, 2-(benzyloxy)ethyl ester | 1.6371 |
| 35 | 4-Ethylbenzoic acid, 2-ethylcyclohexyl ester | 1.5862 |
| 36 | Pyrogallol, 3TMS derivative | 1.5075 |
| 37 | 2-Tetradecanol | 1.4264 |
| 38 | Acetic acid, 3-(5,5-dimethyl-spiro[2.5]oct-4-yl)-1-methyl-propenyl ester | 1.2651 |
| 39 | Metacetamol | 1.1281 |
| 40 | .beta.-Hydroxyquebrachamine | 1.0611 |
| 41 | 2-Methyl-7-phenylindole | 1.0494 |
| 42 | Nonadecane | 1.0001 |
| 43 | 1,3-Dioxane, 4-(hexadecyloxy)-2-pentadecyl- | 0.9728 |
| 44 | 3-Amino-2-phenazinol ditms | 0.8997 |
| 45 | 2-Hexadecanol | 0.8735 |
| 46 | 2-Tridecanone | 0.8565 |
| 47 | Pyruvic acid, 3-hexenyl ester | 0.7915 |
| 48 | 2-Undecanone | 0.7878 |
| 49 | Pentasiloxane, dodecamethyl | 0.7683 |
| 50 | Sulfurous acid, hexyl octyl ester | 0.6353 |
| 51 | Propanoic acid, 2-methyl-, 2-methylpropyl ester | 0.6142 |
| 52 | 3-tert-Butyl-4-hydroxyanisole | 0.6097 |
| 53 | Glutaric acid, dec-2-yl 3,7-dimethyloctyl ester | 0.6064 |
| 54 | Lilial | 0.5886 |
| 55 | 4-Ethylbenzoic acid, 2-pentyl ester | 0.5880 |
| 56 | 2-(Acetoxymethyl)-3-(methoxycarbonyl)biphenylene | 0.5498 |
| 57 | 2-Methyl-7-phenylindole | 0.5223 |
| 58 | m-Camphorene | 0.5018 |
| 59 | 2-Amino-5-methylbenzoic acid | 0.4950 |
| 60 | Benzeneethanol, .alpha.,.alpha.-dimethyl-, acetate | 0.4859 |
| 61 | Glutaric acid, cyclohexylmethyl hept-4-yl ester | 0.4817 |
| 62 | 4-Ethylbenzoic acid, dodec-9-ynyl ester | 0.4594 |
| 63 | Dimethylmalonic acid, isobutyl pentadecyl ester | 0.4415 |
| 64 | Cycloheptanone, 2-benzylidene-, semicarbazone | 0.4372 |
| 65 | Undecanoic acid, ethyl ester | 0.4156 |
| 66 | 4H-Pyran-3-carboxylic acid, 2-amino-5-cyano-6-ethyl-4-(3-pyridinyl)-, methyl ester | 0.3957 |
| 67 | 2'-Hydroxy-5'-methylacetophenone, TMS derivative | 0.3551 |
| 68 | Thymol, TMS derivative | 0.3547 |
| 69 | N-Methyl-1-adamantaneacetamide | 0.3239 |
| 70 | 2-Methyl-7-phenylindole | 0.3143 |
| 71 | 2-Methyl-7-phenylindole | 0.3100 |
| 72 | N-Methyl-1-adamantaneacetamide | 0.2991 |
| 73 | 2-(3,7-Dimethyl-octa-2,6-dienyl)-4-methoxy-phenol | 0.2795 |
| 74 | Hexanoic acid, ethyl ester | 0.2767 |
| 75 | 2'-Hydroxypropiophenone, TMS derivative | 0.2600 |
| 76 | Glutaric acid, monochloride, 4-methylpent-2-yl ester | 0.2587 |
| 77 | 2-(5-Adamantan-1-yl-[1,2,4]oxadiazol-3-yl)-pyridine | 0.2353 |
| 78 | N-Methyl-1-adamantaneacetamide | 0.2263 |
| 79 | (4-Chloro-phenyl)-(1,4-dihydro-benzo[d][1,3]thiazin-2-ylidene)-amine | 0.2183 |
| 80 | 2-Pyridinamine, N-(4,5-dihydro-5-methyl-2-thiazolyl)-3-methyl | 0.2161 |
| 81 | 4-Ethylbenzoic acid, dodec-9-ynyl ester | 0.2120 |
| 82 | 2,4-Di-tert-butylthiophenol | 0.2078 |
| 83 | Acrylophenone, 3,3-diphenyl-, semicarbazone | 0.1982 |
| 84 | Carbonic acid, decyl undecyl ester | 0.1753 |
| 85 | 1-Undecanol | 0.1616 |
| 86 | Pyridine, 3-(5-ethyl-1,2,4-oxadiazol-3-yl)-2-methoxy-6-phenyl- | 0.1560 |
| 87 | Morphinan-6-one, 4,5-epoxy-3,14-dihydroxy-17-(2-propenyl)-, (5.alpha.) | 0.1158 |
| 88 | Aromandendrene | 0.0931 |
| 89 | Longifolene | 0.0881 |
| 90 | (-)-Globulol | 0.0861 |
| 91 | Morphinan-6-one, 4,5-epoxy-3,14-dihydroxy-17-(2-propenyl)-, (5.alpha.) | 0.0786 |
| 92 | Glutaric acid, 2-methylpent-3-yl farnesyl ester | 0.0359 |
| 93 | 4-(2-Bromoethoxy)-3-methoxybenzaldehyde | 0.0282 |
| 94 | Thymoquinone | 0.0239 |
| 95 | 2(3H)-Naphthalenone, 4,4a,5,6,7,8-hexahydro-4,4a-dimethyl-6-(1-methylethenyl) | 0.0096 |

TABLE S10 Fructose content in common fruits.

| **Fruit** | **mg/g DM** |  | **Fruit** | **mg/g DM** |
| --- | --- | --- | --- | --- |
| Apricot | 23.08 |  | Raspberry | 153.75 |
| Pomegranate | 27.50 |  | Sour cherry | 155.22 |
| Pumpkin | 31.54 |  | Cherry | 165.71 |
| Apple, Golden Delicious | 34.62 |  | Currant, black | 185.45 |
| Medlar | 59.39 |  | Melon, Polidor | 206.67 |
| Peach, yellow-green | 61.43 |  | Mulberry, white | 207.14 |
| Fig, wild green | 83.33 |  | Fig, common | 214.44 |
| Nectarine | 95.83 |  | Mulberry, black | 222.22 |
| Quince | 105.60 |  | Melon, honeydew | 234.62 |
| Blackberry | 112.07 |  | Watermelon | 237.00 |
| Plum, red | 116.43 |  | Pear | 247.65 |
| Strawberry, woodland | 120.00 |  | Plum, Ciruela | 249.17 |
| Plum, cherry | 125.88 |  | Grape, white Smederevka | 291.54 |
| Currant, red | 134.71 |  | Apple, Petrovka | 304.62 |
| Apple, Idared | 151.33 |  | Grape, red Vranec | 312.69 |
| Strawberry, common | 153.33 |  | Blueberry | 391.33 |

Adapted from Jovanovic-Malinovska, et al., 2014.


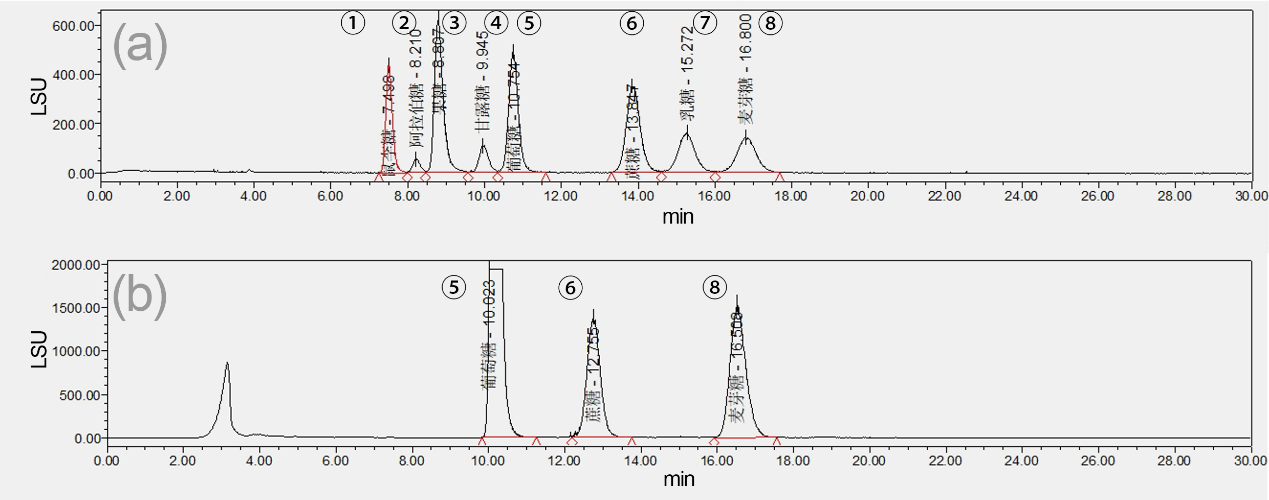


FIGURE S1 (a) HPLC chromatogram of sugar standard. (b) HPLC chromatogram of sugar in the wild diguo fruit (LY01).

①rhamnose, ②arabinose, ③fructose, ④mannose, ⑤glucose, ⑥sucrose, ⑦lactose, ⑧maltose


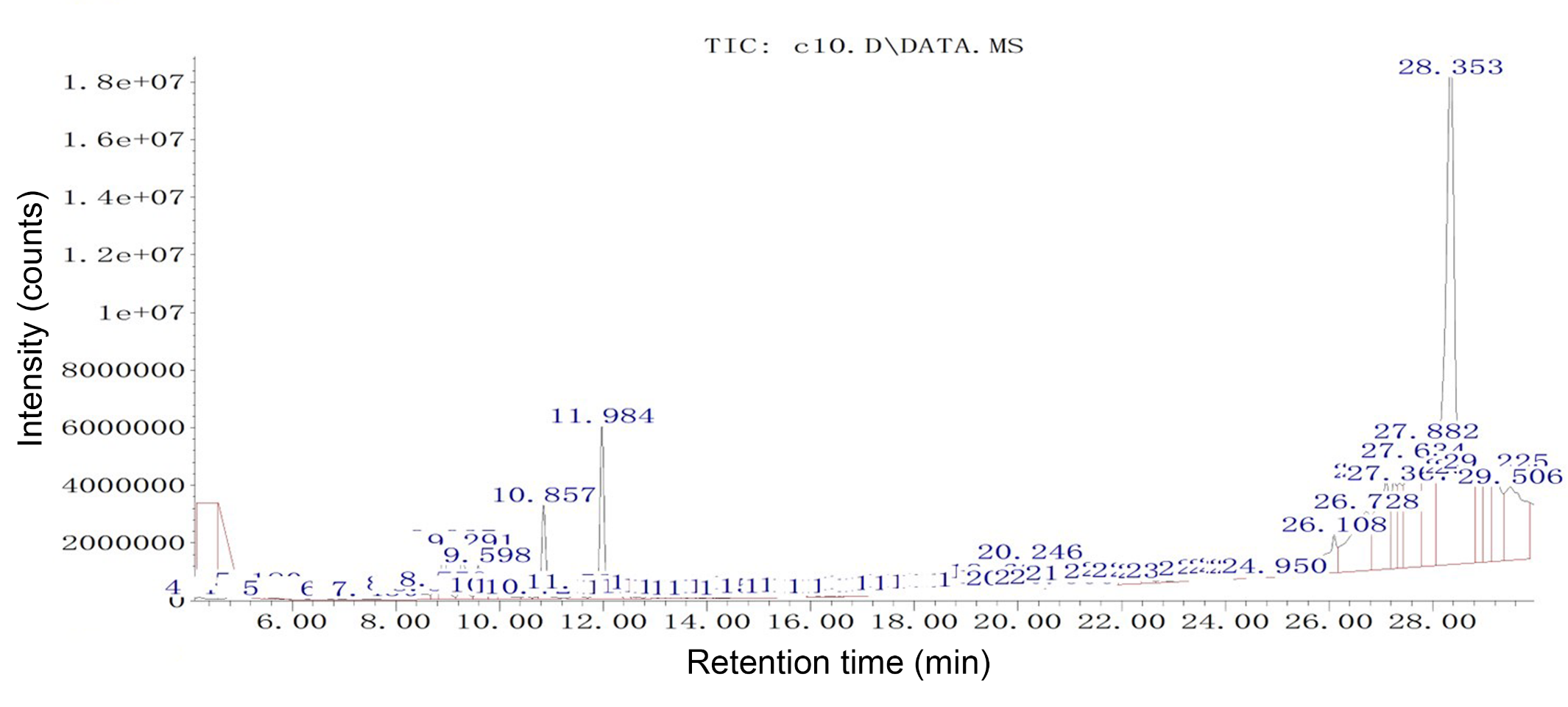


FIGURE S2 Chromatograms of the volatile compounds in the wild diguo fruit.

**SI REFERENCES**

Gong, Y., Luo, W., Chen, H. L., Ren, B., Hu, W. C., & Li, L. M. (2022). Systematical ingredient investigations of *Ficus tikoua* Bur. fruit and immunoregulatory and antioxidant effects of different fractions. *Molecules*, *27*, 6880. https://doi.org/10.3390/molecules27206880

Jovanovic-Malinovska, R., Kuzmanova, S., & Winkelhausen, E. (2014). Oligosaccharide profile in fruits and vegetables as sources of prebiotics and functional foods. *International Journal of Food Properties*, *17*, 949–965. https://doi.org/10.1080/10942912.2012.680221

Shi, D. H., Yang, X. Q., Jiang, H. M., & Liu, Y. (2012). Assessment of nutrients contents and security of *Ficus tikoua*. *Xinan Nongye Xuebao*, *25*, 1398–1401.
